# Near infrared fluorescent imaging of brain tumor with IR780 dye incorporated phospholipid nanoparticles

**DOI:** 10.1186/s12967-016-1115-2

**Published:** 2017-01-23

**Authors:** Shihong Li, Jennifer Johnson, Anderson Peck, Qian Xie

**Affiliations:** 1 Small Animal Imaging Facility, Van Andel Research Institute, Grand Rapids, MI 49503 USA; 2 Center for Cell and Cancer Biology, Van Andel Research Institute, Grand Rapids, MI 49503 USA; 3 Department of Biomedical Science, Quillen College of Medicine, East Tennessee State University, Johnson City, TN 37614 USA; 4 Center for Inflammation, Infectious Disease and Immunity, Quillen College of Medicine, East Tennessee State University, Johnson City, TN 37614 USA

**Keywords:** Near infrared fluorescence imaging, Liposomes, Phospholipid micelles, Brain tumor, Blood–brain barrier

## Abstract

**Background:**

Near-IR fluorescence (NIRF) imaging is becoming a promising approach in preclinical tumor detection and clinical image-guided oncological surgery. While heptamethine cyanine dye IR780 has excellent tumor targeting and imaging potential, its hydrophobic property limits its clinical use. In this study, we developed nanoparticle formulations to facilitate the use of IR780 for fluorescent imaging of malignant brain tumor.

**Methods:**

Self-assembled IR780-liposomes and IR780-phospholipid micelles were prepared and their NIRF properties were characterized. The intracellular accumulation of IR780-nanoparticles in glioma cells were determined using confocal microscopy. The in vivo brain tumor targeting and NIRF imaging capacity of IR780-nanoparticles were evaluated using U87MG glioma ectopic and orthotopic xenograft models and a spontaneous glioma mouse model driven by RAS/RTK activation.

**Results:**

The loading of IR780 into liposomes or phospholipid micelles was efficient. The particle diameter of IR780-liposomes and IR780-phospholipid micelles were 95 and 26 nm, respectively. While stock solutions of each preparation were maintained at ready-to-use condition, the IR780-phospholipid micelles were more stable. In tissue culture cells, IR780-nanoparticles prepared by either method accumulated in mitochondria, however, in animals the IR780-phospholipid micelles showed enhanced intra-tumoral accumulation in U87MG ectopic tumors. Moreover, IR780-phospholipid micelles also showed preferred intracranial tumor accumulation and potent NIRF signal intensity in glioma orthotopic models at a real-time, non-invasive manner.

**Conclusion:**

The IR780-phospholipid micelles demonstrated tumor-specific NIRF imaging capacity in glioma preclinical mouse models, providing great potential for clinical imaging and image-guided surgery of brain tumors.

**Electronic supplementary material:**

The online version of this article (doi:10.1186/s12967-016-1115-2) contains supplementary material, which is available to authorized users.

## Background

Non-invasive imaging modalities, such as computed tomography (CT), magnetic resonance imaging (MRI), single-photon emission computed tomography (SPECT), and positron emission tomography (PET) play key roles in clinical diagnosis, evaluation of disease status and treatment of tumor. The in vivo optical imaging technology using near-infrared fluorescent (NIRF) probes, due to the low NIR absorption and scattering by the tissue, and minimal tissue auto-fluorescence in the NIR window (700–900 nm), is becoming a convenient alternative to the comprehensive imaging modalities in preclinical studies for tumor detection [1–4] and is showing promising results in clinical image-guided oncological surgery [5–10]. The NIRF dye indocyanine green (ICG) has been exploited for imaging of angiogenesis and hepatic segments after hepatectomy [5, 6], as well as for NIR image-guided surgery in a few cancer types [7]. Methylene blue (MB) showed good potential to aid pancreatic tumor resection [8]. Both dyes are approved for clinical use [9, 10]. Among other dyes, NIRF heptamethine cyanine dye IR780 was found to have excellent intrinsic tumor targeting and imaging properties without further modification [11–14], providing great potential for tumor NIRF imaging. IR780′s low cytotoxicity makes it of potential clinical use; however, it is also hydrophobic and insoluble in pharmaceutically acceptable solvents, thus an appropriate formulation is required for clinical use [15, 16]. Several formulations of IR780-encapsulated nanoparticles have been investigated, such as the heparin-folic acid conjugate [17], biodegradable human serum albumin nanoparticles [15], transferrin nanoparticles [16], poly(n-butyl cyanoacrylate) nanocapsules [18], poly(styrene-alt-maleic anhydride)-based diblock copolymer micelles [19], rhenium-188 labeled methoxy poly(ethylene glycol)-block-poly(ε-caprolactone) copolymeric micelles [20], pH-responsive polymeric prodrug micelles [21], phospholipid mimicking homopolymeric micelles [22], bubble-generating folate-targeted liposomes [23], and amsacrine analog-loaded solid lipid nanoparticle [24]. However, most of these carriers were designed for both diagnostic and therapeutic purpose, rarely for fulfilling the unique requirement of NIRF imaging or tumor detection.

**Fig. 1:**
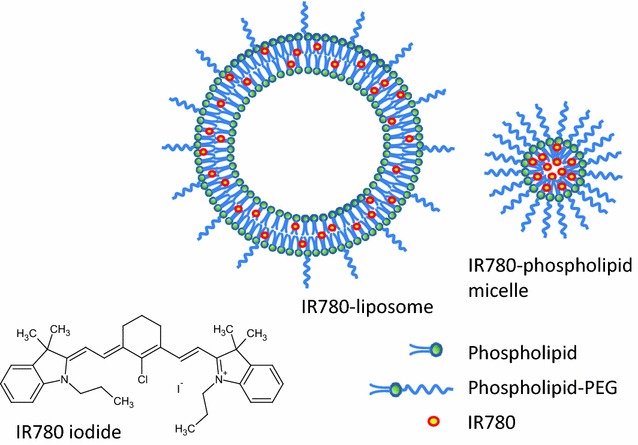
Chemical structure of IR780 and schematic diagram of IR780-liposomes and phospholipid micelles. Phospholipid nanoparticles, including liposomes and micelles are promising drug carriers, which can improve the pharmacokinetic property of the encapsulated drug and its accumulation and retention in solid tumor via the enhanced intratumoral permeability. The liposomes vesicle is composed of a phospholipid bilayer membrane enclosing an aqueous compartment. The phospholipid micelle vesicle has a single layer of phospholipid core with hydrophilic PEG chain coating on the surface. The sizes of phospholipid micelles are generally smaller than liposomes. Hydrophobic IR780 can self-assemble into the phospholipid bilayer membrane of liposomes and the phospholipid core of micelles during the formation of IR780-nanoparticles

Glioblastoma multiforme (GBM) is the most common and lethal primary brain tumor lacking effective therapeutics due to the invasive growth. The migratory tumor cells penetrate into normal parenchyma preventing its complete surgical removal, and its high resistance to chemotherapy and radiotherapy contribute to GBM’s recurrence as a more invasive phenotype [25, 26]. Although it is well established that the degree of surgical resection directly correlated to patient survival [27, 28], most surgery is performed based on the surgeon’s direct visualization of the tumor without any image guidance. Blood–brain barrier (BBB) and blood–tumor barrier (BTB) further challenge the effective treatment of this brain tumor, as most chemotherapy reagents fail to benefit the patients due to the lack of penetration into tumor tissue [29, 30]. NIRF imaging is expected to benefit the preclinical study of GBM and the optical image-guided surgery. Phospholipid nanoparticles, including liposomes and phospholipid micelles are promising drug carriers, which are biocompatible and able to improve the pharmacokinetics of the encapsulated drug and accumulation in a solid tumor via the enhanced permeability and retention (EPR) effect [31–33] In this study, we generated two formulations, liposomes and phospholipid micelles (Schematic diagram in Fig. 1) to incorporate IR780 for in vivo brain tumor imaging using both the human GBM xenograft model and the spontaneous mouse GBM model. The goal was to develop an appropriate formulation of IR780 for pharmaceutically acceptable use for clinical imaging and brain tumor detection.

## Methods

### Chemical and material

1,2-distearoyl-sn-glycero-3-phosphocholine (DSPC) and *N*-(carbonyl-methoxypolyethyleneglycol 2000)-1,2-distearoyl-sn-glycero-3-phosphoethanolamine sodium salt (DSPE-PEG2000) were purchased from NOF America Corporation. Cholesterol, IR780 iodide and other chemicals were purchased from Sigma-Aldrich. Hoechst 33,342, MitoTracker were purchased from Molecular Probes.

### Cell lines and culture

U87MG human glioma cells were from American Type Culture Collection (ATCC, Manassas, VA). U87M2/luc cells were derived from U87MG that stably overexpress firefly luciferase [34]. T98G human glioblastoma cells were from ATCC. All cell lines were grown in DMEM (invitrogen, CA) supplemented with 10% fetal bovine serum (FBS) (Hyclone, UT), 1% penicillin, and 1% streptomycin (invitrogen).

### Preparation of IR780-liposomes and IR780-phospholipid micelles

For IR780-liposome preparation, DSPC, cholesterol and DSPE-PEG2000 at a molar ratio of 54.8:40:5 were dissolved in chloroform (about 15 mg total lipids/ml); 1 mg/ml IR780 iodide in ethanol then was added to final 0.2% molar ratio of dye to total lipids. The solution was rotary evaporated in the dark to dryness in vacuo. The dried lipid film was hydrated in nitrogen gas-flushed 0.9% saline to a final total lipid concentration of 30 mM, vortexed and ultra-sonicated under a nitrogen atmosphere for final suspension of the lipid particles. The suspension was repeatedly extruded 16 times through a 100 nm Whatman Nucleopore track-etched polycarbonate membrane at 56 °C. The acquired clear bluish suspension was flushed with nitrogen gas, sealed in a glass vial and stored at either 4 or −20 °C in the dark.

To prepare IR780-phospholipid micelles, thin lipid film composed of DSPC and DSPE-PEG2000 containing IR780 (molar ratio, 59.5:40:0.5) was formed in the same way as for the liposomes preparation, then hydrated with nitrogen gas-flushed 0.9% saline to a final total lipid concentration of 15 mM, vortexed and ultra-sonicated at 56 °C for 15 min under a nitrogen gas atmosphere, and extruded through a 100 nm Whatman Nucleopore track-etched polycarbonate membrane. The acquired clear cyan micelle suspension was stored under the same conditions as the liposomes.

### Characterization of IR780-liposomes and IR780-phospholipid micelles

#### Particle sizing of IR780-liposomes and IR780-phospholipid micelles

The particle size distributions of IR780-liposomes and IR780-phospholipid micelles were measured with a DynaPro dynamic light-scattering system (Wyatt Technology, CA). Before measurement, the samples were diluted with 200 nm membrane-filtered saline to reach appropriate signal concentrations.

#### Near infrared absorption and fluorescence spectra of IR780-liposomes and IR780-phospholipid micelles

Visible absorption spectra of IR780, IR780-liposomes and IR780-phospholipid micelles diluted in PBS, ethanol/PBS and PBS/FBS mixtures were measured by a preconfigured UV–visible spectrometer (StellarNet, Inc., FL). The near infrared fluorescent spectra were measured using a Synergy™ Neo HTS Multi-mode microplate reader (Bio-Tek, VT).

#### Stability of IR780-liposomes and IR780-phospholipid micelles

IR780-liposomes and IR780-phospholipid micelles were aliquoted as stock solution (20×) and kept in the dark at 4 or −20 °C. At different time points the aliquots were equilibrated at room temperature and further diluted with PBS into a working solution (1×). The NIR fluorescent signal intensity was measured at Ex/Em = 745/815 nm. The stability was determined using relative fluorescent intensity (FI/FI_0_) which was measured by fluorescent signal intensity of samples stored at 4 °C (FI) as compared with those stored at −20 °C (FI_0_).

### In vitro cellular uptake of IR780-liposomes and IR780-phospholipid micelles

T98G and U87MG cells were pre-cultured in a flask with DMEM medium supplied with 10% FBS at 37 °C with 5% CO_2_. For cellular uptake experiments, cells were trypsinized and re-seeded on glass coverslips at a density of 6 × 10^4^/cm^2^ and cultured for another 24 h to reach 60–80% confluency; the medium was replaced with fresh medium supplied with free IR780 (IR780 stock solution in ethanol freshly diluted in PBS), IR780-liposomes or IR780-phospholipid micelles with the final IR780 concentration at 1 μM IR780. After 30 min of incubation, the cells were washed twice with PBS and supplied with fresh DMEM medium without phenol red followed by further staining with Hoechst 33,342 (10 μg/ml) for 1 h and MitoTracker (1 nM) for 10 min. Microscopic images of cells washed with cold PBS and then supplemented with medium were acquired using a confocal laser scanning microscope (Nikon A1 Plus-RSi, Japan). The excitation/emission wavelengths for fluorescent imaging of Hoechst 33,342, IR780 and Mitotracker were 350/461, 650/780 and 554/576 nm, respectively.

### GBM tumor models

All studies involve animals were approved by the VARI Institutional Animal Care and Use Committee (IACUC). To establish U87M2/luc ectopic xenograft tumor model, 5 × 10^5^ cells in 100 μl of PBS were subcutaneously inoculated into the flank region of 6-week old nude mice to initiate tumor growth. Three weeks after inoculation, tumor initiation rate reached 90%. Tumor size was measured twice a week using a caliper with tumor volume (mm^3^) = width^2^ × length/2. The ability of IR780-nanoparticles to cross the BBB was tested using the U87M2/luc orthotopic model as previously described [34]. Briefly, mice were inoculated using a stereotaxic frame. A burr hole was created through the skull 2 mm posterior to the bregma, 2 mm anterior to the central suture, and 3 mm below the meninges; U87M2/luc cells (5 × 10^5^ cells in 5 μl of PBS) were injected into the brain parenchyma. The orthotopic tumor growth was measured by bioluminescence signal intensity (BLI). Each mouse received an intraperitoneal injection of 100 μl of 30 mg/ml D-luciferin sodium solution, and images were taken after 10 min using an AMI1000 optical imager (Spectral Instruments Imaging, Inc., Tucson, AZ). To induce mouse glioma, plasmids pT2/C-Luc/PGK-SB100, pT/CAGGS-NRASV12, pKT2/CLP-AKT, and pT2/shP53/GFP4 (provided by Dr. David Largaespada, University of Minnesota) were mixed and the intracerebroventricular injection was performed as previously described [35]. In brief, neonatal mice were placed on ice for 4 min to induce anesthesia before being secured in a cooled, “neonatal rat” stereotaxic frame (Stoelting, IL) maintained at 4–8 °C by a dry ice/ethanol reservoir. A 10 μl syringe fitted with a 30 gauge hypodermic needle (12.5° bevel; Hamilton, NV) attached to an automatic injector (Stoelting, IL) was used to inject plasmids at a flow rate of 0.7 μl/min into the right lateral cerebral ventricle. A total of 2 μg of plasmid DNA (mixed at 1:1:1:1) in 2 μl was injected into each mouse to induce spontaneous glioma. No incision was made for the injection. The skull of a neonate was penetrated with the needle for all injections. Growth of the orthotopic tumor was monitored by BLI as described above.

### NIRF imaging with IR780 incorporated nanoparticles in GBM mouse models

Fourteen nude mice bearing U87M2/luc tumors with volumes of 122–580 mm^3^ were divided into three groups based on balanced tumor volumes: (1) free IR780 (IR780 freshly prepared in ethanol/saline (V/V, 1:9) (n = 4), (2) IR780-liposomes, and (3) IR780-micelles (IR780-liposomes or IR780-phospholipid micelles freshly diluted in saline containing 2 nmol of IR780, n = 5). Each mouse was intravenously injected via tail vein with 100 μl imaging agent. The sequential whole body NIRF images at different time points (5 min, 1, 4, 24, 48, 72, and 96 h post injection) were acquired using an AMI1000 optical imager (Ex/Em = 745/810 nm, acquisition time: 1 s). At two time points, 24 h and 96 h post injection of IR780 agents, bioluminescent images also were acquired (imaging acquisition time: 10 s) to determine tumor growth.

Five nude mice with four bearing U87M2/luc orthotopic tumors and one tumor-free healthy control, and three *FVB* mice bearing spontaneous GBM induced by activation of RAS and AKT and *Trp53* loss (*FVB/NRAS/AKT/shP53*) [35] were used to evaluate the imaging capacity of IR780-phospholipid micelles in orthotopic brain tumors. With orthotopic tumors, each mouse was intravenously injected with 100 μl IR780-phospholipid micelles freshly diluted in saline containing 4 nmol of IR780 (U87M2/luc orthotopic model) or 6 nmol of IR780 (*FVB/NRAS/AKT/shP53* spontaneous tumor model). The sequential whole body images at different time points were captured following the same procedure performed for U87M2/luc ectopic models.

### Ex vivo imaging and histological staining

At the end of the in vivo imaging, mice bearing tumors were sacrificed, and brain, heart, lung, liver, spleen, stomach, intestine, normal muscle and skin from lumbar were dissected for immediate fluorescent photography using the AMI1000 optical imager. For histology analysis, mice brains were harvested and fixed in 10% neutral-buffered formalin and embedded into paraffin blocks and slides (20 μm) were cut for H&E staining. For microscopic NIRF images, additional brain sections were cut from the paraffin blocks. Unstained slides were scanned using the Odyssey imager (LI-COR Biosciences) and software suite version 3. Settings were optimized for the highest resolution and power to allow for visualization of both the fluorescent target and non-fluorescent anatomy. Resolution was set to 21 microns with no focal offset and excitation intensity was set to 6.0 for the 800 nm channel only.

### Statistical analysis

All experimental data were shown as mean ± SD unless stated otherwise. Comparisons of data between 2 groups or among 3 groups were analyzed using independent-samples *t* test or one-way analysis of variance (ANOVA) at P < 0.05.

## Results

### Particle sizes of IR780-liposomes and IR780-phospholipid micelles

The hydrodynamic diameter of freshly prepared IR780-liposomes was 95.1 ± 2.2 nm with a polydispersion index (PI) = 0.078. The hydrodynamic diameter of freshly prepared IR780-phospholipid micelles was 26.4 ± 8.2 nm with PI = 0.096 (Additional file 1: Figure S1). The average particle size of IR780-liposomes and IR780-phospholipid micelles were not significantly changed after stored at 4 °C in dark over a period of 40 days.

### NIR absorption and fluorescence properties of IR780-liposomes and IR780-phospholipid micelles

The maximum NIR absorption wavelength of IR780 in ethanol was at 783 nm (Additional file 1: Figure S2). Dilution of the IR780 stock solution with water resulted in the hypochromatic shift of the absorption band and decreased absorbance, probably due to IR780 aggregation or the molecular stacking of aromatic ring structure in aqueous solution, which can be reversed by adding ethanol. The insertion of IR780 into liposomes or phospholipid micelles caused the bathochromic shift of the absorption band and decreased absorbance (791 nm for IR780-liposomes and 793 nm for IR780-phospholipid micelles (Additional file 1: Figure S2). Adding ethanol to IR780-liposomes or IR780-phospholipid micelles resulted in maximal NIR absorption shifted to 783 nm, indicating the release of IR780 from dissolved nanoparticles. Both IR780-liposomes and IR780-phospholipid micelles showed broad NIR fluorescent spectrum with maximum excitation/emission wavelength at 745/815 nm. (Additional file 1: Figure S3).

### Stability of IR780-liposomes and IR780-phospholipid micelles

**Fig. 2:**
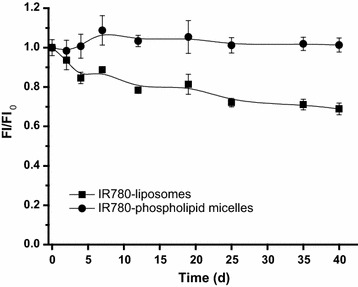
Relative fluorescent intensity of IR780-liposomes and IR780-phospholipid micelles. FI/FI_0_ was measured by fluorescence signal intensity of samples stored in the dark at 4 °C as compared with those stored in the dark at −20 °C (n = 3, Ex/Em = 745/815 nm). *Short bar* refers to standard deviation

The long term stability of IR780-liposomes and IR780-phospholipid micelles was evaluated using fluorescence signal intensity of samples stored at 4 °C (FI) as compared with that stored at −20 °C (FI_0_) in dark (Fig. 2). While both IR780-liposomes and IR780-phospholipid micelles showed constant FI_0_ over the 40-day period, IR780-liposomes showed decreased FI with time, resulting in a significant reduced FI/FI_0_ = 0.69 at day 40. In contrast, IR780-phospholipid micelles showed stable FI/FI_0_ = 1.0 during the entire 40 days, demonstrating their superior stability as compared with IR780-liposomes.

### In vitro cellular uptake of IR780-liposomes and IR780-phospholipid micelles

**Fig. 3:**
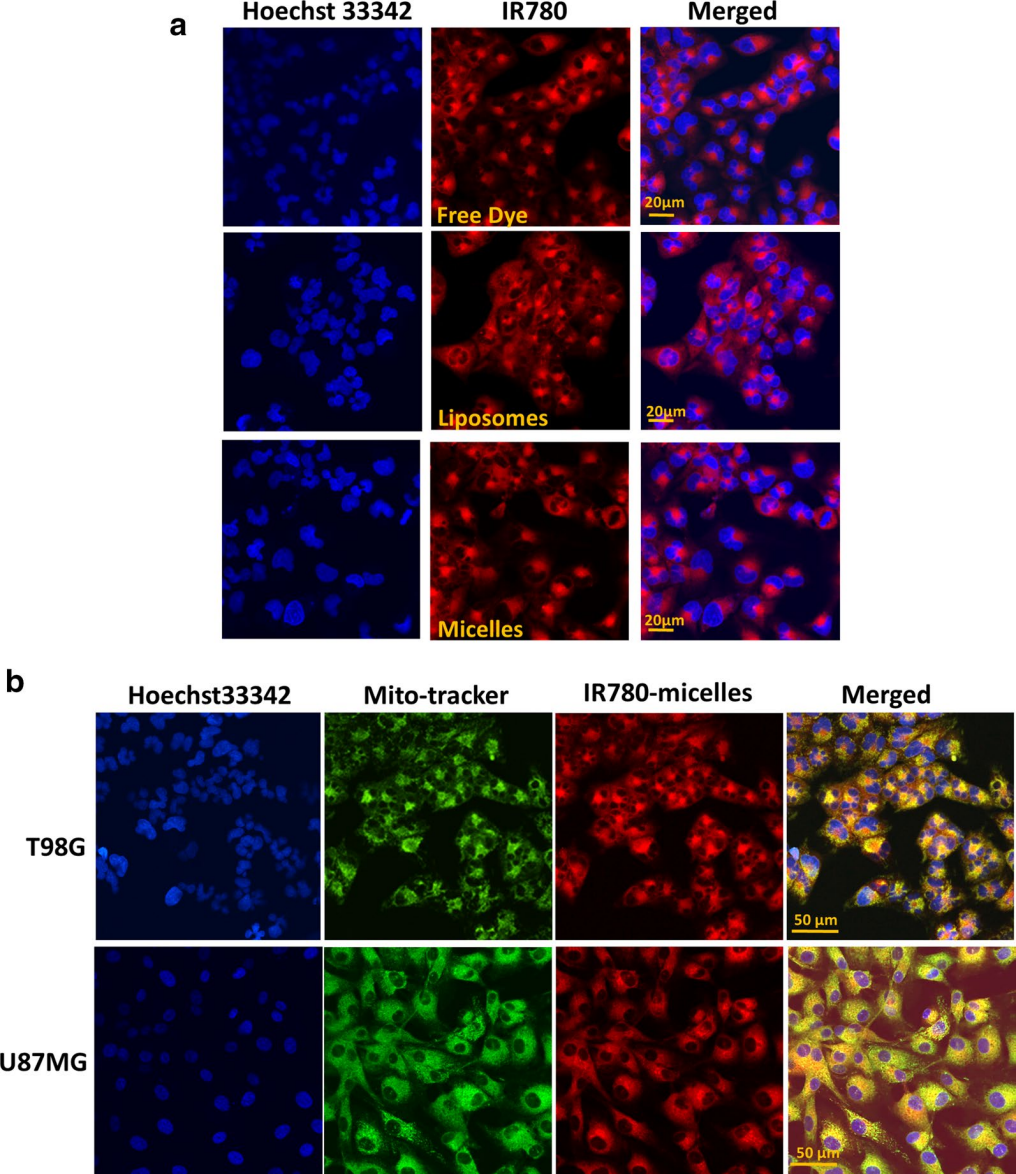
Mitochondrial localization of IR780 by confocal microscopy. **a** T98G cells were cultured with different formulations of IR780 for 30 min followed by Hoechst 33342 staining. **b** T98G and U87MG cells cultured with different formulations of IR780 and dual-stained with Hoechst33342 and MitoTracker

We first compared intracellular uptake of free IR780, IR780-liposomes, and IR780-phospholipid micelles using T98G cells (Fig. 3). All three formulations showed the same perinuclear cytoplasmic accumulation after a 30-min incubation with the cells, and also suggested a mitochondrial localization (Fig. 3a). To confirm, T98G and U87MG cells cultured with IR780-micelles also were dual-stained with Mitotracker (a specific mitochondrial marker) and Hoechst 33,342 (a specific nuclear marker) followed by confocol microscopy (Fig. 3b). We observed strong overlapping signals of IR780-micelles and Mitotracker (Fig. 3b, merged), indicating a mitochondrial accumulation of IR780-micelles, which is consistent to previous reports that preferential accumulation of free IR780 dye and liposomal IR780 were found in mitochondria of multiple tumor cells [11, 12, 36]. Therefore, the incorporation of IR780 with liposomes or phospholipid micelles preserved the same mitochondrial retention feature as free IR780.

### Preferential uptake and retention of IR780-nanoparticles in U87M2/luc ectopic tumors

**Fig. 4:**
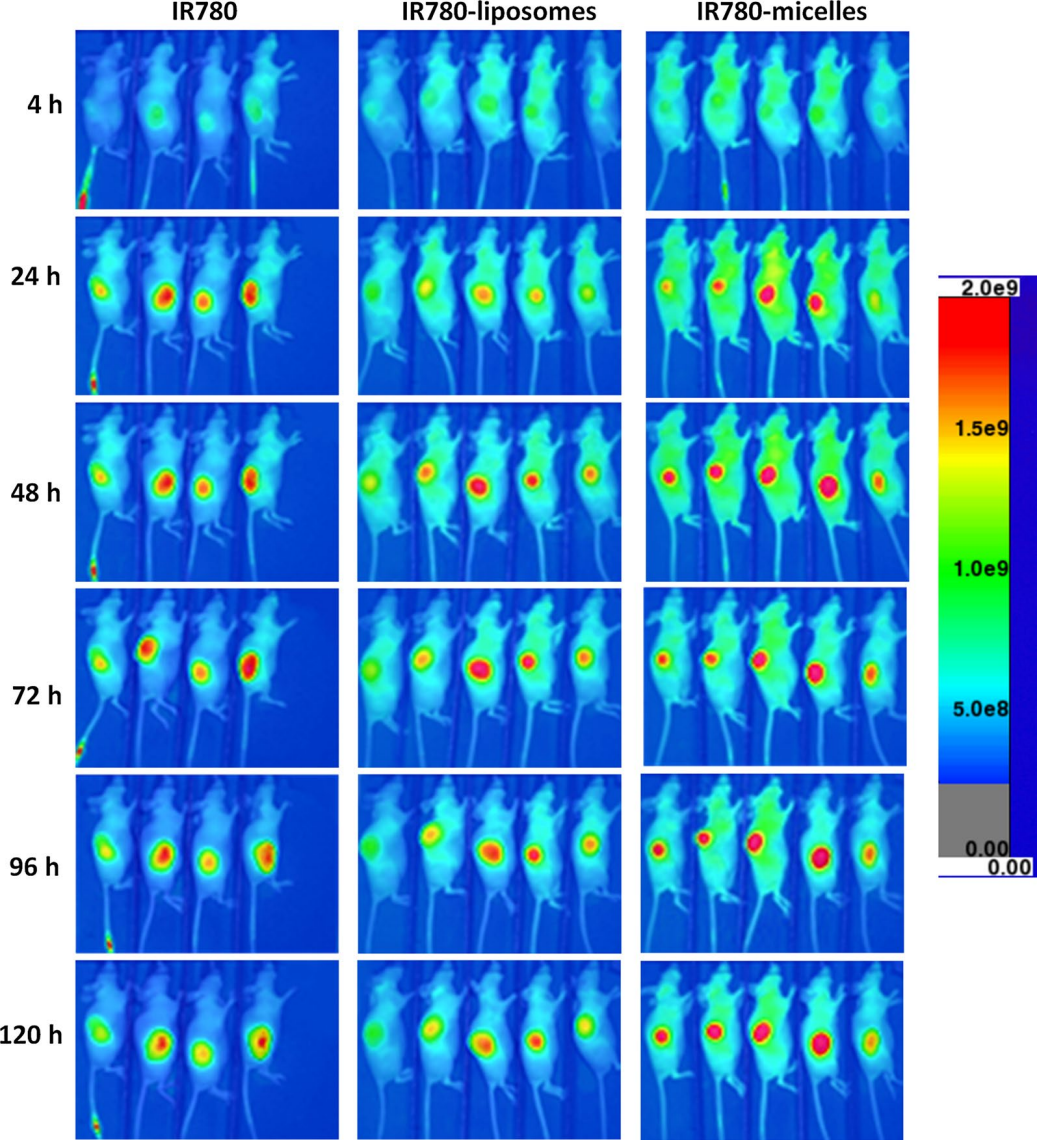
In vivo NIRF imaging of IR780-nanoparticles with U87M2/luc ectopic model. Nude mice bearing U87MG subcutaneous tumors of similar size were dosed with free IR780, IR780-liposomes, or IR780-phospholipid micelles via tail vein injection. Post injection, sequential NIRF images were taken at each time point as indicated using the AMI1000 optical imager at Ex/Em = 745/810 nm

The in vivo tumor-specific uptake of free IR780, IR780-liposomes, and IR780-phospholipid micelles were evaluated using the U87M2/luc ectopic tumor model (Fig. 4). The tumors were barely visualized at 4 h post drug administration, however, were clearly delineated at 24–120 h in all three groups. The tumors from IR780-phospholipid micelles group showed stronger NIRF signals than those from IR780-liposomes and free IR780 groups, both of which displayed similar NIRF intensities at each time point (Additional file 1: Figure S4), demonstrating that phospholipid micelle encapsulation is the best formulation for IR780 for tumor-specific targeting and retention.

The ex vivo NIRF images taken after the mice were euthanized at the end of day 5 further confirmed the major accumulation of IR780 in tumors regardless of formulation (Additional file 1: Figure S5). Minor accumulation of IR780 was found in liver, lung, skin and kidneys.

### Real-time NIRF imaging with IR780-phospholipid micelles in GBM orthotopic models

**Fig. 5:**
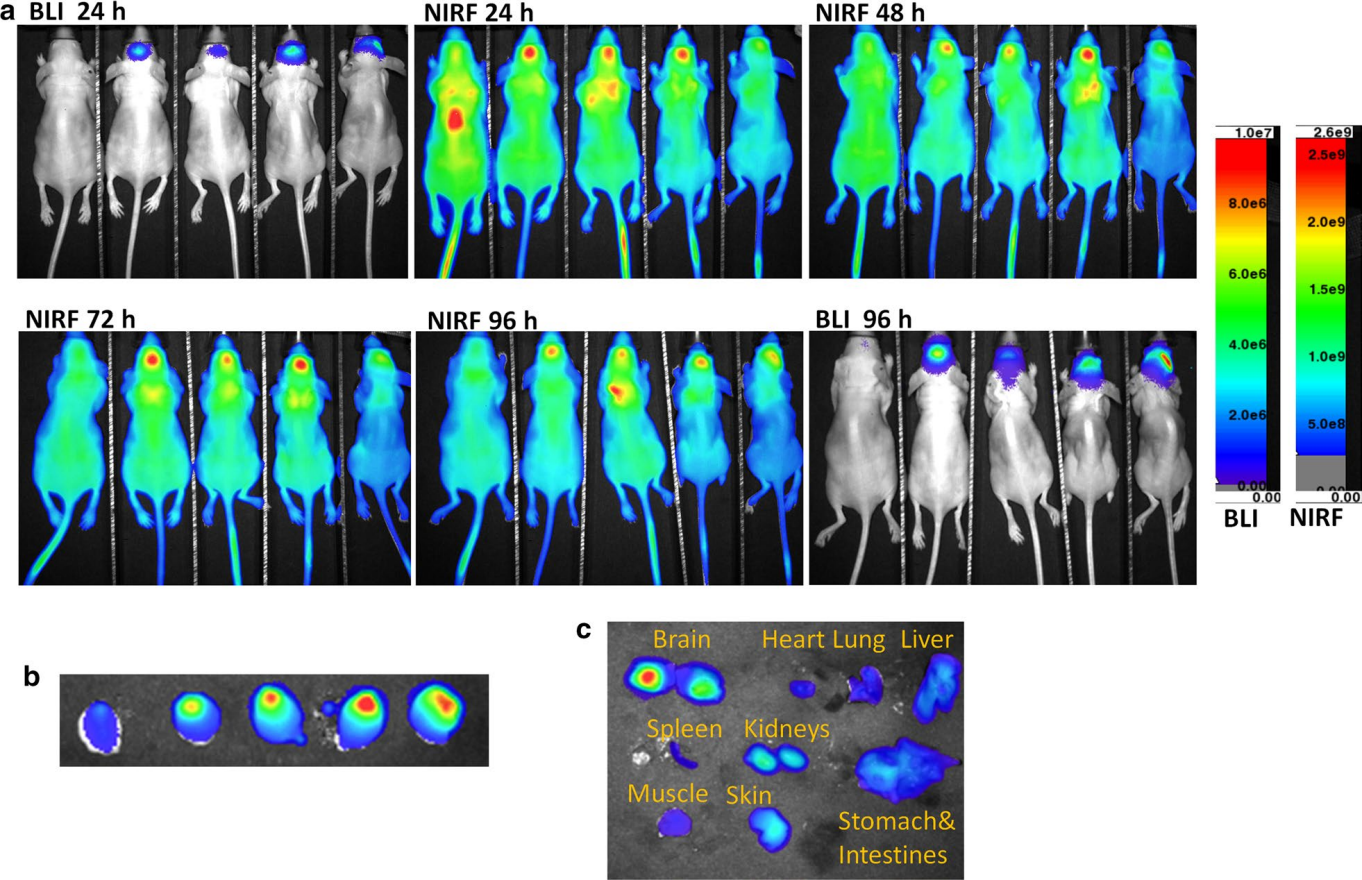
In vivo NIRF imaging of IR780-phospholipid micelles with the U87M2/luc orthotopic model. **a** Nude mice bearing U87M2/luc orthotopic tumors were dosed with IR780-phospholipid micelles via tail vein injection. Post injection, sequential BLI and NIRF images were taken at each time point as indicated using the AMI1000 optical imager at Ex/Em = 745/810 nm. Note that the healthy control mouse (the 1st one on the *left*) did not show any signals. **b** NIRF image of dissected mouse brains after the last whole body imaging. **c** Representative NIRF image of dissected brain and other organs from a mouse bearing U87M2/luc orthotopic tumor. *Upper* brain, lung, heart and liver; *Middle* spleen, kidneys, stomach and intestines; *Lower* muscle, skin

The blood–brain barrier is a natural challenge for drug delivery to brain tumors. Because IR780-micelles showed the best intratumoral uptake, we further evaluated its NIRF imaging capacity using the U87M2/luc orthotpic model (Fig. 5). After tumor growth was confirmed by bioluminescent imaging, IR780-phospholipid micelles were injected via tail vein and NIRF images were acquired every 24 h for 4 days. Strong fluorescent signal intensity with high contrast was observed in mice brain 24 h post injection and lasted for at least 4 days, in the same area where emitting the bioluminescent signal, demonstrating a good intracranial tumor accumulation and retention. In contrast, the healthy control mouse did not show significant NIRF signal in normal brain. The tumor-specific targeting of IR780-micelles was further confirmed by the ex vivo imaging with dissected brains and other organs at time of necropsy (Fig. 5b, c). The NIRF signal was mainly concentrated in the brain tumors rather than surrounding normal tissues. Compared to tumor-bearing brain, skin and kidneys had minor retention of the dye, and other dissected organs only showed faint signals, indicating a low retention of IR780-phospholipid micelles (Fig. 5c).

**Fig. 6:**
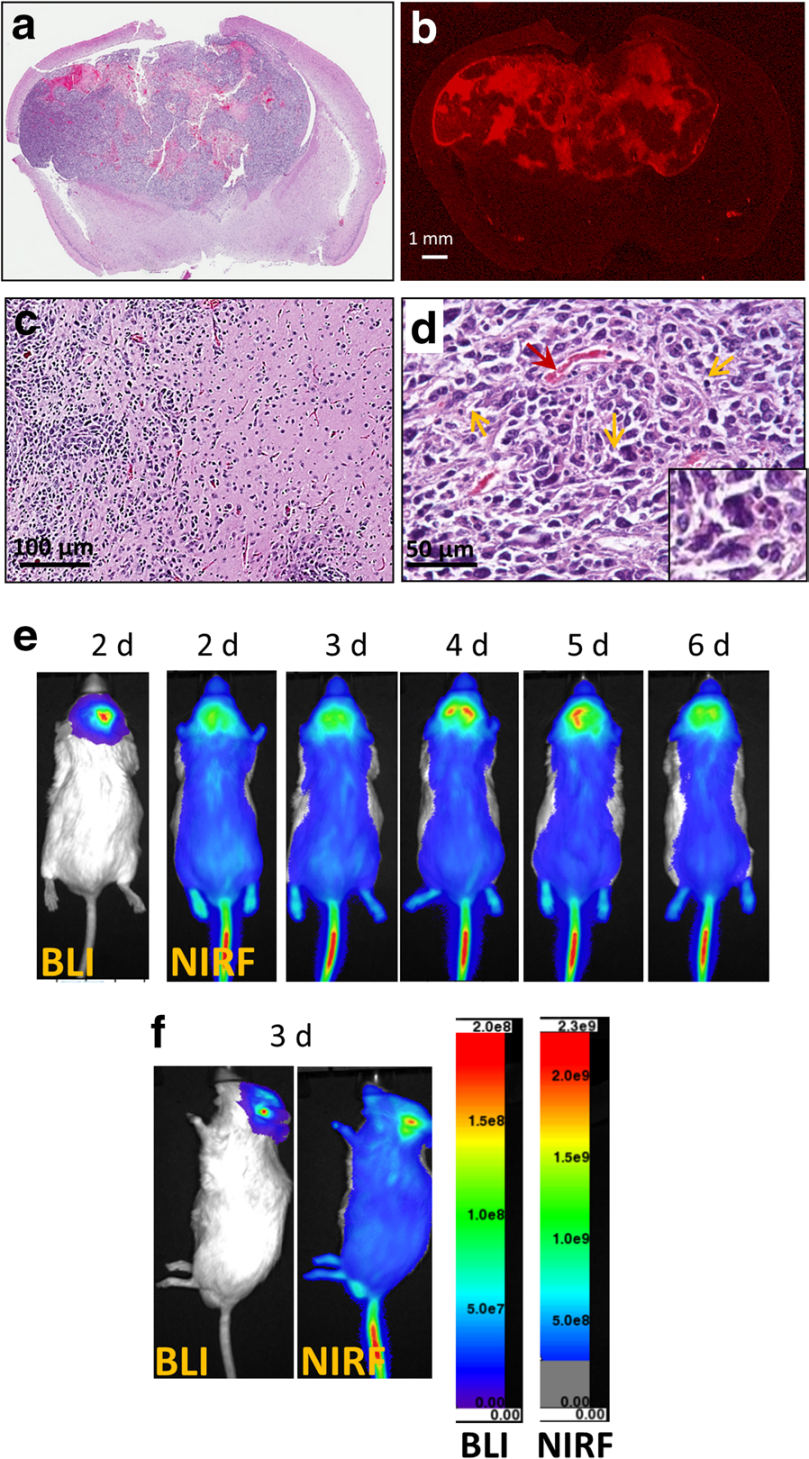
Real-time NIRF imaging of IR780-phospholipid micelles using the GBM spontaneous mouse model. **a** Representative GBM tumor section showing invasive tumor growth in H&E staining. **b** A duplicate unstained brain section showing tumor targeting of IR780-phospholipid micelles by NIRF microscopic imaging. GBM hallmarks including invasive tumor growth penetrating into normal brain (**c**), and anaplastic nuclear (*arrowed yellow* and insert) and glomeruloid body-like vascular structure (*arrowed red*, **d**). **e** Whole-body bioluminescent and NIRF imaging of a tumor-bearing mouse at different times post injection of IR780-phospholipid micelles. **f** Intratumoral accumulation of IR780-micelles at day 3 post injection as imaged in the lateral position. Note that bioluminescence and fluorescence signals come from same area

GBM is the most malignant brain tumor due to the extensive invasiveness; activation of RAS and AKT via receptor tyrosine kinases (RTK) pathways frequently occurs in GBM patients [37]. With the *Sleeping Beauty* transposon system, Wiesner et al. demonstrated that dysregulation of the RAS/RTK pathway induced spontaneous GBM formation in mouse; embedding luciferase as a reporter gene further allows tumor growth be monitored using BLI [35]. We therefore also tested the NIRF imaging capacity of IR780-phospholipid micelles using this mouse model. We show that overexpression of NRAS and AKT induced invasive tumor growth as reported previously (Fig. 6a–c) [35]. Abnormal vasculature (Fig. 6d) and anaplastic cell division (Fig. 6d arrows and insert) were also observed, indicating a fast growing phenotype. From the same mouse, a 6-day consecutive bioluminescent and fluorescent imaging was acquired after IR780-phospholipid micelles were injected through tail vein (Fig. 6e, f). We show that the NIRF signal with high contrast started to show up in the brain on day 2, gradually reached peak on day 4, followed by remission thereafter until the animal was euthanized on day 6 (Fig. 6e). From the side view at day 3, the fluorescent and bioluminescent imaging clearly showed co-localization of the signals (Fig. 6f), demonstrating a tumor-specific accumulation and retention of IR780-phospholipid micelles. Consistent to the in vivo imaging results, NIRF imaging of brain sections using un-stained paraffin slides showed strong fluorescent signal intensity enriched in tumor area (Fig. 6b), supporting that IR780-phospholipid micelles can penetrate BBB and accumulate in brain tumors.

## Discussion

Molecular imaging focuses on the noninvasive quantitation and real-time visualization of molecular processes as they occur in vivo, and NIRF imaging is becoming an attractive approach for tumor detection in preclinical animal studies and image-guided resection in clinical oncological surgery. The hydrophobic IR780 iodide is a promising tumor-specific targeting NIRF probe [12, 13, 38]. Because IR780 is a weak positively-charged lipophilic molecule, we envisioned it to intimately interact with the weak anionic phosphate moiety of phospholipid and therefore can be stably packaged within the phospholipid nanoparticles (Fig. 1) during their self-assembled formation. To develop an appropriate formulation for biomedical use of IR780, we formulated IR780-liposomes and phospholipid micelles each carrying 0.2 molar % and 0.5 molar % of IR780. Higher molar ratios of dye were avoided to prevent the severe fluorescent quenching upon aggregation of dye inside the nanoparticles. Both preparations were homogeneously dispersed suspensions and showed narrow particle size distribution; the particle size of IR780-phospholipid micelles was much smaller than IR780-liposomes (26.4 ± 8.2 versus 95.1 ± 2.2 nm), indicating a better potential to cross the BBB and BTB. Notably, the insertion of lipophilic IR780 into liposomes or phospholipid micelles reached 100% during the preparation and required no further purification step. Comparing with IR780-liposomes, which gradually lost NIRF signal intensity over time, IR780-micelles were very stable, and therefore, encapsulation into micelles is a better formulation for IR780. In addition to phospholipids, cholesterol also serves as a major lipid component which facilitates the liquid-ordered structures and stabilizes the bilayer of the liposome structure [39, 40]. We therefore tested the compatibility of free IR780 dye with the phospholipid and cholesterol in aqueous suspension, respectively. While DSPC strongly adsorbed free IR780 from aqueous suspension after severe vortexing and centrifugation, cholesterol showed no adsorption ability with free IR780. This incompatibility of cholesterol with IR780 may be the cause of the lower stability of IR780-liposomes compared to IR780-micelles.

GBM is the most devastating brain tumor invading normal brain tissue and escaping surgical eradication; most chemotherapeutic reagents lack of permeability across the BBB and BTB, further limiting the options of effective therapeutics. Developing nanoparticle-incorporated NIRF dyes not only may improve the brain tumor imaging for tumor detection, but also can aid GBM surgery in the operation room to define the invasive boarder. Among the NIRF probes that are under development, ICG and MB are FDA approved for clinical use, although both are blood pool agents that are not specific for any tumor tissue [3]. In contrast, IR780 has intrinsic tumor-targeting activity [13, 14, 41], and the potential to work as a sonosensitizer for sonodynamic therapy in cancer, suggesting its dual-role as both an imaging probe and an anti-cancer reagent [42]. Using T98G and U87MG cells, we demonstrate that IR780-liposomes and IR780-phospholipid micelles acted the same as free IR780 that mechanistically functions through mitochondrial (Fig. 3). The 5-day NIRF imaging using the U87M2/luc ectopic model further verified the preferential uptake and accumulation of IR780-liposomes and IR780-phospholipid micelles in brain tumor, where the micelles displayed relatively stronger IR780 intratumoral accumulation and retention. Moreover, no significant uptake and accumulation of IR780-phospholipid micelles was observed in brain tissues from normal (Fig. 5 and Additional file 1: Figure S6) or ectopic tumor-bearing mice Fig. (4), indicating normal brain is not a targeting organ of IR780.

While the BBB is a natural challenge for drug delivery to brain tumors, our results show that IR780-phospholipid micelles could efficiently reach U87M2/luc orthotopic tumors 24 h post injection. Potent fluorescent signal intensity was found mainly at the tumor area, indicating a specific tumor targeting and accumulation (Fig. 5). The NIRF imaging with the spontaneous GBM mouse model (Fig. 6) showed consistent results to that with the U87M2/luc orthotopic model, further demonstrating that IR780-phospholipid micelles could penetrate the BBB and BTB in targeting invasive GBM.

While the results from our preclinical studies have demonstrated that IR780-phospholipid micelles, combining the passive tumor targeting feature of nanoparticles via EPR effect [33] and the intrinsic tumor-targeting feature of IR780, is a promising candidate nanomedicine for tumor imaging and image-guided surgery, there are also challenges prevent its clinical translation. Compared to UV and visible light, NIR light has its advantage by deep penetration in biological tissues, however, the skin penetration remains a major challenge. Recent studies suggest that NIRF may penetrate skin, but the optical NIRF signal can be affected by the different skin components [43, 44]. With ICG, a NIRF dye shares similar optical property to IR780, the depth of penetration for NIR fluorescence is estimated to be between 2 and 4 cm and could be improved if the noise floor of devices could be reduced [45]. Thus, NIRF imaging using IR780-micelles will have similar limitations for detecting glioma and other types of cancer, whereas further studies addressing NIRF light absorption, scattering, transmission and reflection in context of different skin components are required to improve its clinical translation. Developing NIRF instrumentations with optimized light source and detectors can further enhance the capacity of NIRF as a non-invasive imaging modality in clinics. Unlike mouse skull, human skull is more solid and tumors are more in depth. As IR780 is expected to have limitation in penetration through the skull, it will not be a good clinical option for non-invasively imaging brain tumor patients, although it remains a valuable tool for imaging pre-clinical intracranial tumor models. However, such a disadvantage does not reduce its potential value to facilitate invasive tumor imaging and clinical image-guided oncological surgery. NIRF imaging can be used to indicate invasive margin during brain tumor resection, or to identify local invasion and metastatic lesions for other types of cancer.

## Conclusion

In summary, we prepared IR780 carrying liposomes and phospholipid micelles and investigated their potential as biomedically acceptable agents for brain tumor imaging. The IR780-phospholipid micelles were more stable than the IR780-liposomes. Both nanoparticles showed good NIRF imaging with the GBM ectopic xenograft model. Moreover, the IR780-phospholipid micelles also showed preferential uptake in the human GBM orthotopic mouse model and the spontaneous mouse GBM model, demonstrating good preclinical and clinical potential for sensitively imaging brain tumors and other types of cancer. In the future, it is worthwhile to further explore the NIRF imaging capacity of IR780-phospholipid micelles in detecting small and early stage tumors in different cancer types, by which individual mouse bearing small ectopic and orthotopic tumors are to be imaged for evaluation.

## Additional file

**Additional file 1.** **Figure S1**. Histograms of the hydrodynamic size distributions of IR780 encapsulated nanoparticles. **Figure S2**. NIR absorption spectra of IR780, IR780-liposomes and IR780-phospholipid micelles. **Figure S3**. NIR fluorescent spectra of IR780-liposomes and IR780-phospholipid micelles. **Figure S4**. In vivo NIRF signal intensity of IR780-nanoparticles in U87MG ectopic tumors. **Figure S5**. Representative Ex vivo NIRF images of dissected organs from U87MG ectopic tumor bearing mice. **Figure S6**. Ex vivo NIRF images of organs and tissues from healthy control mice.

## Abbreviations

BBB: blood–brain barrier
BLI: bioluminescence signal intensity
BTB: blood–tumor barrier
CT: computed tomography
DSPC: 1,2-distearoyl-sn-glycero-3-phosphocholine
(DSPE-PEG2000): *N*-(Carbonyl-methoxypolyethyleneglycol 2000)-1,2-distearoyl-sn-glycero-3-phosphoethanolamine sodium salt
EPR: enhanced permeability and retention
GBM: glioblastoma multiforme
ICG: indocyanine green
MB: methylene blue
MRI: magnetic resonance imaging
NIRF: near-infrared fluorescent
PET: positron emission tomography
SPECT: single photon emission computed tomography
RTK: receptor tyrosine kinase
