# Supplementary material for: Near infrared fluorescent imaging of brain tumor with IR780 dye incorporated phospholipid nanoparticles
Source: J Transl Med. 2017 Jan 23;15:18. doi: 10.1186/s12967-016-1115-2 (PMC5260002; doi:10.1186/s12967-016-1115-2)
Supplement: Supplementary file 1 — Additional file 1. Figure S1. Histograms of the hydrodynamic size distributions of IR780 encapsulated nanoparticles. Figure S2. NIR absorption spectra of IR780, IR780-liposomes and IR780-phospholipid micelles. Figure S3. NIR fluorescent spectra of IR780-liposomes and IR780-phospholipid micelles. Figure S4. In vivo NIRF signal intensity of IR780-nanoparticles in U87MG ectopic tumors. Figure S5. Representative Ex vivo NIRF images of dissected organs from U87MG ectopic tumor bearing mice. Figure S6. Ex vivo NIRF images of organs and tissues from healthy control mice. [file 12967_2016_1115_MOESM1_ESM.docx]

**Additional File 1**

**Near infrared fluorescent imaging of brain tumor with IR780 dye incorporated phospholipid nanoparticles**

Shihong Li^1^, Jennifer Johnson^2^, Anderson Peck^1^, and Qian Xie^2,3*^

^1^Small Animal Imaging Facility, Van Andel Research Institute, Grand Rapids, MI 49503

^2^Center for Cell and Cancer Biology, Van Andel Research Institute, Grand Rapids, MI 49503

^3^Department of Biomedical Science, Quillen College of Medicine, East Tennessee State University, Johnson City TN 37604

**Files in this Data Supplement:**

Supplementary Figures 1-6

Supplementary Figure 1. Histograms of the hydrodynamic size distributions of IR780 encapsulated nanoparticles.

Supplementary Figure 2. NIR absorption spectra of IR780, IR780-liposomes and IR780-phospholipid micelles.

Supplementary Figure 3. NIR fluorescent spectra of IR780-liposomes and IR780-phospholipid micelles.

Supplementary Figure 4. *In vivo* NIRF signal intensity of IR780-nanoparticles in U87MG ectopic tumors.

Supplementary Figure 5. Representative *Ex vivo* NIRF images of dissected organs from U87MG ectopic tumor bearing mice.

Supplementary Figure 6. *Ex vivo* NIRF images of organs and tissues from healthy control mice.

**Supplementary Figures**

**
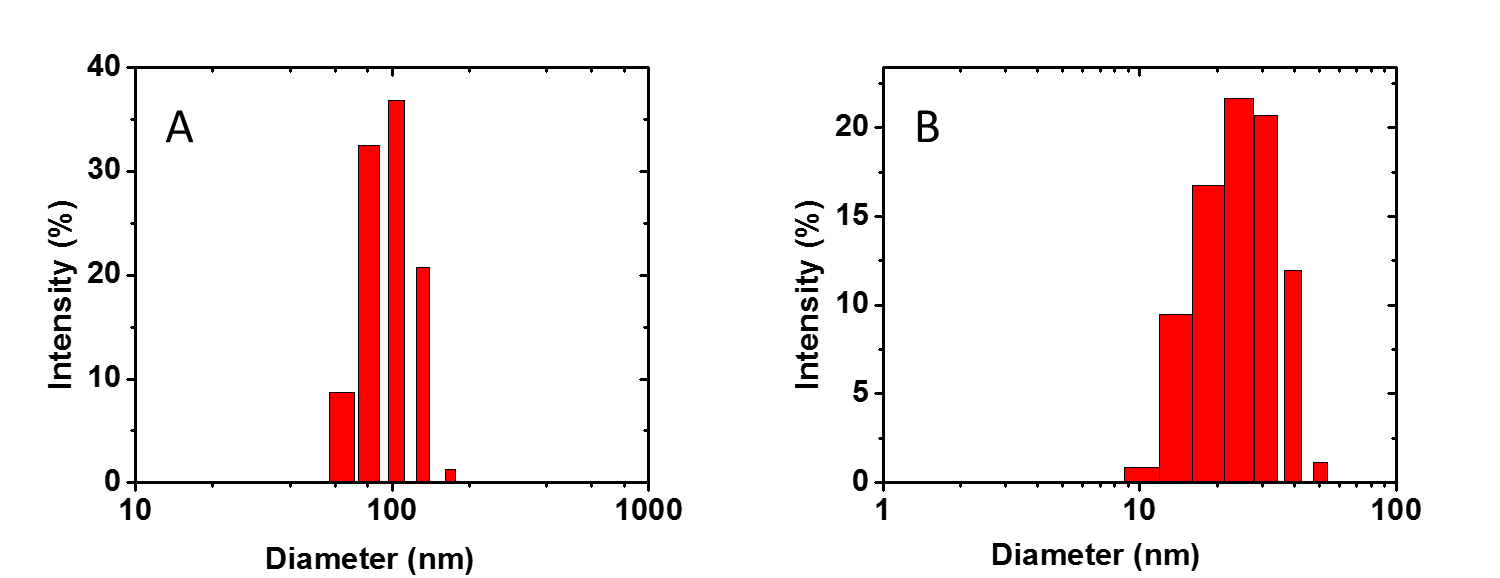
**

**Figure S1.** Histograms of the hydrodynamic size distributions of IR780-encapsulated nanoparticles.

(A) IR780-liposomes containing 0.2 molar % IR780

(B) IR780-phospholipid micelles containing 0.5 molar % IR780.

**Figure S2.** NIR absorption spectra of IR780, IR780-liposomes and IR780-phospholipid micelles.
Stock solutions of IR780 in ethanol, IR780-liposomes and IR780-phospholipid micelles were diluted in saline (V/V, 1:250, 1:20 and 1:70, respectively) and measured by a preconfigured UV-visible spectrometer.

**Figure S3.** NIR fluorescent spectra of IR780-liposomes and IR780-phospholipid micelles.
IR780-liposomes and IR780-phospholipid micelles containing 2 mM and 1 mM of total lipids, respectively. The fluorescent signal intensity was recorded at individual wavelength as indicated.

**Figure S4.** *In vivo* NIRF signal intensity with the U87MG ectopic mouse model.

Nude mice bearing U87MG subcutaneous tumors of similar size were dosed with free IR780, IR780-liposomes, or IR780-phospholipid micelles, via tail vein injection. Sequential NIRF images were taken at each post injection time point as indicated using AMI-100 optical imager at Ex/Em=745 nm/810 nm (see Figure 4). Fluorescence signal intensity of each tumor was calculated using AMIView software.

* Represents significant difference by ANOVA analysis at 0.05 level. Short bar refers to standard deviation.

**
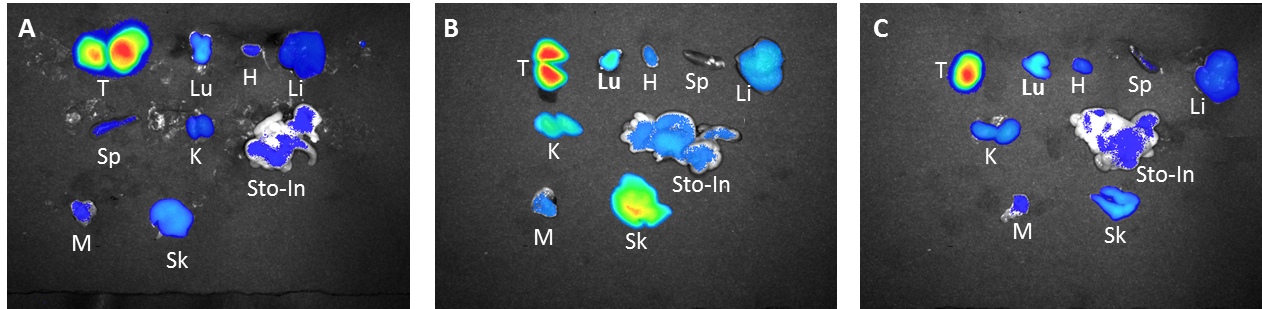
**

**Figure S5.** Representative *ex vivo* NIRF images of dissected organs from U87MG ectopic tumor-bearing mice.

Nude mice bearing U87MG ectopic tumors were euthanized at 120 h post-injection of free IR780 (A), IR780-liposomes (B), or IR780-phospholipid micelles (C). Dissected organs (Legend: T, tumor; Lu, lung; H, heart; Li, liver; Sp, spleen; K, kidneys; Sto-In, stomach and intestines; M, muscle; Sk, skin.) were collected and imaged using the AMI-100 optical imager at Ex/Em = 745 nm/810 nm.

**
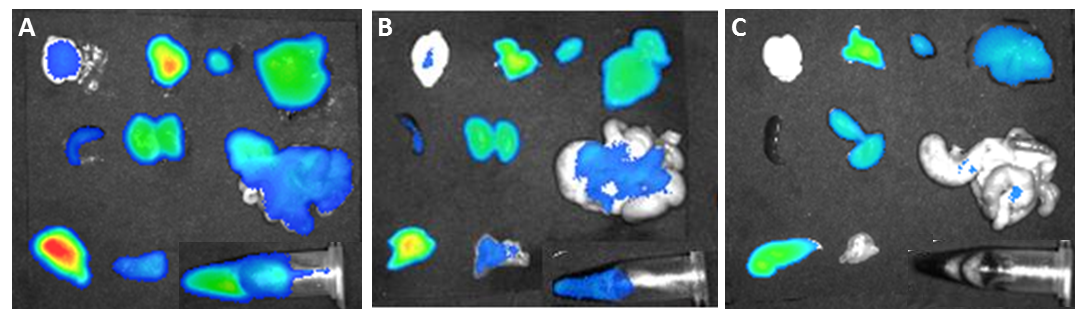
**

**Figure S6.** *Ex vivo* NIRF images of organs and tissues from healthy control mice.

Healthy nude mice were euthanized at 24 h (A), 48 h (B), and 72 h (C) post-injection of IR780-phospholipid micelles. Dissected organs (Upper: brain, lung, heart and liver; Middle: spleen, kidneys, stomach and intestines; Lower: skin, muscle and blood sample collected in PE centrifugal tube) were collected and imaged using AMI-100 optical imager at Ex/Em = 745nm/810nm.
